# Mechanistic Insights into Milk Minerals Driving Bone Development and Mineralization in Growing Rats

**DOI:** 10.3390/nu18152569

**Published:** 2026-08-06

**Authors:** Yile Peng, Yalin Zhou, Simon Bøge Riis, Jing Yin, Muke Han, Zhang Wen, Wanyun Ye, Xudong Liu, Weiwei Shi, Xuezeng Wang, Jiahui Luo, Yajun Xu

**Affiliations:** 1 Department of Nutrition and Food Hygiene, School of Public Health, Peking University, No. 38 Xueyuan Road, Beijing 100083, China; 1810306134@pku.edu.cn (Y.P.);; 2 Arla Food Ingredients Group P/S, Sønderhøj 10-12, 8260 Viby J, Denmark

**Keywords:** milk minerals, bone mineral density, mineralization, gut microbiota

## Abstract

Objective: To investigate the effect of milk minerals on bone mineral density (BMD) and bone quality in growing rats and explore the underlying mechanisms related to calcium absorption, bone metabolism, and the gut–bone axis. Methods: Sixty healthy 4-week-old male Sprague-Dawley (SD) rats were randomly divided to five groups based on their body weight: Low-Calcium Control Group (Control), Low-Dose milk mineral Group (Low), Medium-Dose milk mineral Group (Medium), High-Dose milk mineral Group (High) and Calcium Carbonate Control Group (CaCO_3_), which received the same dose level (elemental calcium) as the High group. The milk mineral dosage was set at 5, 10, and 15 times the human recommended intake of elemental calcium. After 12 weeks of intervention, femurs were collected for analysis of BMD, bone microstructure, and bone mechanical strength. Additionally, analyses included calcium levels in the femur, feces, and diet; serum bone metabolism biomarkers; tissue protein expression; as well as gut microbiota composition and short-chain fatty acid content. Result: Milk mineral exhibited non-inferior efficacy to CaCO_3_ in increasing femoral calcium content, enhancing BMD, and improving bone microarchitecture. Notably, the Medium group achieved comparable bone-protective effects to the CaCO_3_ group despite a 20.8% lower calcium content, which was accompanied by a relatively high calcium absorption rate (90.9% vs. 86.5%). With respect to serum markers, milk mineral maintained bone formation while suppressing bone resorption, resulting in a net anabolic state comparable to that of CaCO_3_. Milk mineral significantly upregulated the protein expression of renal CYP27B1 and intestinal calcium ion transporters, and increased serum IGF-I levels. Furthermore, milk mineral promoted the enrichment of certain specific gut microbial genera, which showed a significant positive correlation with IGF-I, bone calcium content and BMD. Conclusions: Milk mineral supplementation appears to promote bone formation and mineralization in growing rats, accompanied by enhanced intestinal calcium absorption, enrichment of characteristic gut microbes and elevated microbial metabolite concentrations.

## 1. Introduction

Bone mineral density (BMD) is a critical indicator of overall skeletal health. Maintaining healthy BMD is essential for preserving physical stability and mobility. The clinical significance of BMD is profoundly evident in osteoporosis, a major global public health issue. Research data indicate that osteoporosis affects approximately 200 million people worldwide. It is projected that the number of patients will increase by 23% over the next decade, posing a significant challenge to healthcare systems worldwide [1,2,3]. Osteoporosis is characterized by reduced bone mass and microarchitectural deterioration of bone tissue. Consequently, BMD, which serves as a quantitative measure of bone mass, has been established by the World Health Organization (WHO) as the primary diagnostic criterion for the disease [4]. Peak bone mass (PBM) refers to the highest level of BMD and bone mass achieved during the growth period. Bone mass is a key determinant of osteoporosis and fragility fractures. Epidemiological studies have shown that a 10% increase in population PBM is associated with a 50% reduction in the risk of fractures later in life [5]. Although standardized reference values for PBM have not yet been established, and the precise age of its attainment remains unclear [6], considerable evidence indicates that the period before age 30 is a critical window for bone mass accumulation. Subsequently, bone mass typically undergoes a gradual decline thereafter [7]. Therefore, effectively increasing bone mass before the age of 30, particularly during the growth and development period, to achieve a higher peak bone mass is crucial for the prevention of osteoporosis and its associated complications.

Calcium is a cornerstone nutrient for both the prevention and management of osteoporosis, and calcium supplementation represents a well-established strategy for enhancing PBM [8]. A variety of calcium sources are available, including inorganic calcium, calcium derived from milk, algal calcium, and calcium citrate. Numerous investigations have consistently demonstrated that milk-derived calcium is at least non-inferior to inorganic calcium in improving BMD and enhancing calcium bioavailability [9,10,11,12,13]. It is noteworthy that dairy products can contribute 50–60% of the daily calcium intake [14]. Moreover, dairy products provide phosphorus, magnesium, and proteins—phosphorus co-crystallizes with calcium as hydroxyapatite, magnesium serves as an essential cofactor for vitamin D metabolism and bone crystal stabilization, and proteins form the organic matrix of bone. Some fortified varieties also provide vitamin D, which aids in calcium absorption and bone metabolism. Additionally, milk-derived bioactive peptides such as casein phosphopeptides can chelate calcium ions and maintain their solubility in the intestinal lumen, thereby facilitating calcium absorption. Such inherent nutrient interactions may confer favorable skeletal benefits for dairy consumption [14]. As a concentrated extract of bovine milk, milk mineral concentrate (MMC) retains these natural bone-supporting nutrients. In contrast, calcium carbonate, the most widely used calcium supplement and a standard comparator for evaluating calcium bioavailability, is a single inorganic calcium source without these additional components. This multi-nutrient synergy may partly explain the possible differential bone-related outcomes between MMC and pure calcium carbonate. However, existing research [9,10,11,12,13] has primarily documented the positive effects of milk mineral on bone mass, while the differential efficacy of milk mineral versus calcium carbonate in improving BMD remains unconfirmed, and the underlying molecular mechanisms are poorly characterized. Furthermore, some studies [9,12,15] have focused solely on the effects of milk mineral on BMD in adulthood or later life, rather than the critical developmental stage of active bone accretion. Therefore, this study aims to investigate whether milk mineral exerts different effects from calcium carbonate on BMD during the growth and development stage, as well as the potential molecular mechanisms of milk mineral involved, thereby providing a theoretical foundation and novel nutritional solutions for improving skeletal health.

## 2. Materials and Methods

### 2.1. Test Substance and Experimental Animals

A natural MMC was obtained from Arla Food Ingredients Group P/S, Viby J, Denmark (Capolac^®^ MM-0525 BG, Aarhus, Denmark). This product is manufactured via membrane filtration of raw bovine milk. The concentrate contains 27% calcium, 12.5% phosphorus, 8% lactose, and 3% milk protein, with the remainder consisting of crystal water and mineral ash. Notably, the milk protein fraction originates from endogenous casein and whey peptides naturally retained during the milk filtration process, rather than externally supplemented protein isolates.

Sixty healthy, 4-week-old male Sprague-Dawley rats weighing 60–75 g were obtained from the Experimental Animal Center of Peking University Health Science Center (Animal Certificate No. SCXK (Jing) 2022-0037). The animals were housed in a specific pathogen-free (SPF) barrier facility under controlled conditions: temperature maintained at 22 ± 2 °C, relative humidity at 50–60%, and a 12 h light/12 h dark cycle. Throughout the experimental period, the rats were allowed ad libitum access to food and water. This study was reviewed and approved by the Biomedical Ethics Committee of Peking University. All experimental procedures complied with the requirements of animal welfare and ethics, with ethical approval number: PUIRB-LA2023309.

### 2.2. Animal Grouping and Treatment

#### 2.2.1. Animal Grouping and Supplement Administration

Following a 3-day acclimatization period, the rats were weighed to record their initial body weight and randomly assigned into five groups (*n* = 12 per group) based on their weight: Low-Calcium Control Group (Control), Low-Dose MMC Group (Low), Medium-Dose MMC Group (Medium), High-Dose MMC Group (High) and Calcium Carbonate Control Group (CaCO_3_). The CaCO_3_ group received an equivalent dose of elemental calcium to that administered to the High-Dose MMC group.

Doses were set at 5, 10, and 15 times the recommended dietary allowance of calcium for humans. This calculation was based on an adult calcium recommendation of 800 mg/d and a standard body weight of 60 kg. Given the high basal metabolic rate and rapid clearance of test substances in rats, gradient high-dose interventions were established to ensure sufficient calcium exposure in bone tissue, and the highest-dose group can be used to preliminarily explore the upper effective range and safety margin. The MMC for each dosage group and the CaCO_3_ group were prepared by thoroughly mixing them into a basal low-calcium diet (calcium content: 150 mg/100 g of diet; the composition of the low-calcium basal diet is presented in Table 1; Ke’ao Xieli (Tianjin) Feed Co., Ltd., Tianjin, China). The calcium dosage (mg/kg body weight) was converted to a concentration in the feed (mg/100 g feed), calculated based on a rat feed intake of 8.0 g/100 g body weight.

All rats were allowed ad libitum access to the formulated diets and deionized water (to eliminate exogenous calcium intake from drinking water). The experimental feeding period lasted 12 weeks. Upon completion of the feeding trial, blood samples were collected from the femoral artery and centrifuged to isolate serum. Following cervical dislocation, the rats were dissected to harvest femurs, tibias, jejuna, ilea, colons and colonic contents. All collected samples were stored immediately at −80 °C for subsequent biochemical and molecular analysis.

#### 2.2.2. Calcium Metabolism Experiment

To compare the differences in calcium absorption rates among groups, a calcium metabolism study was conducted following 3 weeks of experimental feeding. The rats were individually housed in metabolic cages for a 1-day adaptation period. During the subsequent 3 consecutive days (72 h total), fecal samples were collected. The daily food intake for each animal was recorded to calculate the actual calcium intake. The calcium absorption rate was calculated using the following formulas:

Calcium Intake(mg/d) = Calcium content in feed (mg/g) × Food consumption (g/d)

Fecal Calcium (mg/d) = Calcium content in feces (mg/g) × Fecal output (g/d)

$$\mathrm{Calcium}\;\mathrm{Absorption}\;\mathrm{Rate}(\%)=\frac{\mathrm{Calcium}\;\mathrm{Intake}-\mathrm{Fecal}\;\mathrm{Calcium}}{\mathrm{Calcium}\;\mathrm{Intake}}\;\times \;100\%$$

### 2.3. Measured Parameters

#### 2.3.1. Body Weight, Body Length, and Food Intake of Experimental Animals

Body weight of the rats was measured weekly using an electronic balance, and body length was measured with a tape measure. Weekly food intake was also recorded to calculate average daily food intake.

#### 2.3.2. Femur Size and BMD

Total length of the right femur was measured using a precision caliper (YBKC-100, Mietek, San Jose, CA, USA). A transverse line was marked at the femoral midshaft to define the mid-point measurement site; the femoral neck was used as the proximal measurement site; and the lowest point of the articular groove at the distal end was designated as the distal measurement site. BMD at the femoral midshaft, proximal and distal ends was measured using Dual-energy X-ray Absorptiometry (DXA; Parameter 3D, KUBTEC, Stratford, CT, USA). Prior to measurement, the instrument was calibrated with a standard bone phantom to ensure that the relative measurement error compared with the standard value was within ±3%.

#### 2.3.3. Femur Microstructure

Following DXA analysis, the microstructures of trabecular and cortical bone in the femur were evaluated using micro-CT Scanner (μCT; SkyScan 1276, Bruker, Billerica, MA, USA). The distal metaphysis of the right femur was scanned. The distal femur metaphysis was scanned with a pixel size of 8.062 μm. All scans were performed at a projection resolution of 1452 × 1372 pixels with a rotational step of 0.4° and a total range of 180°. The volume of interest (VOI) for trabecular bone was defined as a region starting 1.5 mm below the distal femoral growth plate, and the VOI for cortical bone was defined as a region beginning 4 mm below the femur distal growth plate. A total of 200 consecutive slices were analyzed for the trabecular bone and cortical bone. Trabecular bone structural parameters included bone volume fraction (BV/TV), trabecular thickness (Tb.Th), trabecular number (Tb.N), trabecular separation (Tb.Sp), connectivity density (Conn.D) and structure model index (SMI). Cortical bone microstructural parameters comprised cortical tissue mineral density (Ct.TMD) and cortical thickness (Ct.Th).

#### 2.3.4. Femur Biomechanics

Following μCT scanning, a three-point bending mechanical test (WDW-5, Jinan Dongce Testing Machine Technology Co., Ltd., Jinan, China) was performed on the right femur to evaluate its ultimate load (N, defined as the maximum force sustained before fracture, reflecting bone strength) and ultimate displacement (mm, defined as the maximum deformation prior to fracture, indicating bone toughness). Each femoral specimen was positioned on two supports spaced 15 mm apart, with the femur allowed to bend about the mediolateral axis. A load was applied to the midspan of the bone at a constant deformation rate of 2.5 mm/min until fracture occurred. The stress–strain curve was recorded throughout the procedure and parameters including ultimate load and ultimate deformation were extracted from the curve.

#### 2.3.5. Determination of Femur Dry Weight and Calcium Content in Femur, Feces, and Feed

Following the three-point bending test, the right femur of each rat was dried to a constant weight in an oven at 105 °C (Heratherm, Thermo Fisher Scientific, Waltham, MA, USA) to obtain the femoral dry weight. Fecal and feed samples were similarly dried to constant weight at 105 °C. A 0.3–1.0 g aliquot of each dried sample was accurately weighed and digested in 15–20 mL of a mixed-acid solution (nitric acid:perchloric acid = 4:1). The mixture was heated on an electric hot plate until white fumes appeared and digestion was continued until the solution became colorless. Deionized water was added to the colorless digestate, and the solution was boiled to remove residual acids; this step was repeated to ensure the final volume did not exceed 1 mL. A blank control was processed concurrently throughout the digestion procedure by adding an equivalent volume of the mixed-acid solution without any sample and subjecting it to the same treatment. Calcium content in bone, feces, and feed was ultimately quantified by ICP-MS (8900, Agilent Technology, Inc., Santa Clara, CA, USA).

#### 2.3.6. Serum Biochemical Indicators

Serum biochemical indicators were measured using a commercially available ELISA kits: Serum Calcium (Ca) and Phosphorus (P) (Jiangsu Aidisheng Biological Technology Co., Ltd, Yancheng, China), Parathyroid hormone (PTH), Calcitonin (CT), 25-(OH)D_3_, Procollagen Type I N-Terminal Propeptide (PINP), C-terminal telopeptide of type I collagen (CTX-I) and Insulin-like Growth Factor-1 (IGF-I) (Jiangsu mmbio Industrial Co., Ltd., Yancheng, China).

#### 2.3.7. Western Blot Analysis

Western blot was conducted to determine the protein expression levels of Receptor Activator of Nuclear Factor Kappa-B Ligand (RANKL) and Osteoprotegerin (OPG) in the left tibia, Cytochrome P450 Family 27 Subfamily B Member 1 (CYP27B1) in the kidneys, and Transient Receptor Potential Cation Channel Subfamily V Member 6 (TRPV6), Calbindin-D9k (CaBP-D9k), Plasma Membrane Calcium ATPase 1b (PMCA1b), NCK Adaptor Protein 1 (NCK1), Claudin-2 (CLDN2), and the vitamin D receptor (VDR) in each segment of intestinal tissue.

Frozen tissue samples (approximately 50 mg each) were homogenized in 1 mL of RIPA (R0010, Beijing Solarbio Science & Technology Co., Ltd., Beijing, China) lysis buffer on ice using a mortar and pestle. The homogenate was transferred to a 1.5 mL microcentrifuge tube, incubated on ice for 30 min, and then centrifuged at 12,000 rpm for 30 min at 4 °C. The supernatant was collected, and protein concentration was determined using a bicinchoninic acid (BCA; P0011, Beyotime Biotechnology, Shanghai, China) protein assay kit according to the manufacturer’s instructions.

Protein samples were prepared for electrophoresis and subjected to Western blot analysis. Briefly, proteins were separated by SDS-PAGE using a 10% separating gel and a stacking gel, followed by electrophoretic transfer onto a polyvinylidene difluoride (PVDF; Lot#0000219088, Merck-Millipore, Burlington, MA, USA) membrane. The membrane was blocked with 5% non-fat milk in TBST (PS103, Shanghai Epizyme Biomedical Technology Co., Ltd., Shanghai, China) for 1 h at room temperature (or overnight at 4 °C), then incubated overnight at 4 °C with primary antibodies (Wuhan ABclonal Biotechnology Co., Ltd., Wuhan, China, Abcam Ltd., Cambridge, UK and Cell Signaling Technology, Inc., Boston, MA, USA) diluted in blocking solution. After washing three times with TBST (10 min each), the membrane was incubated with the corresponding horseradish peroxidase (HRP; LF101, Shanghai Epizyme Biomedical Technology Co., Ltd., Shanghai, China)-conjugated secondary antibody for 1 h at room temperature. Following another series of TBST washes, protein bands were visualized using an enhanced chemiluminescence (ECL; SQ201L, Shanghai Epizyme Biomedical Technology Co., Ltd., Shanghai, China) detection system and imaged with a chemiluminescence imaging system (Universal Hood II, Bio-Rad Laboratories, Hercules, CA, USA). Band intensity was quantified using Image J software (version 1.54f).

#### 2.3.8. Gut Microbiota and SCFAs Analysis

The composition and relative abundance of gut microbiota, as well as the concentrations of their metabolic product short-chain fatty acids (SCFAs), were analyzed using 16S rDNA sequencing and GC-MS.

Microbial genomic DNA was extracted using the TIANamp Stool DNA Kit (TIANGEN Biotech (Beijing) Co., Ltd., Beijing, China) according to the manufacturer’s instructions. The hypervariable V3–V4 region of the bacterial 16S rRNA gene was amplified via PCR with universal primers 341F and 805R, using Phusion High-Fidelity DNA Polymerase (F530S, Thermo-Fisher Scientific, Waltham, MA, USA). The resulting amplicons were purified, quantified, and sequenced on an Illumina NovaSeq 6000 (NovaSeq 6000, Illumina Inc., San Diego, CA, USA) platform (paired-end, 2 × 250 bp) by a commercial service provider. Raw sequencing data were processed using QIIME 2 (version 2024.10). Sequences were demultiplexed, quality-filtered, denoised, merged, and chimera-removed using the DADA2 plugin to generate amplicon sequence variants (ASVs). Taxonomic assignment was performed against the SILVA reference database (version 138) using a naive Bayes classifier with a confidence threshold of 0.7. α diversity (Chao1, Shannon, and Simpson indices) and β diversity (Bray–Curtis dissimilarity) were calculated. Principal coordinates analysis (PCoA) based on Bray–Curtis distance and permutational multivariate analysis of variance (PERMANOVA) were performed to assess overall microbial community differences among groups. Linear discriminant analysis effect size (LEfSe) was employed to identify differentially abundant taxa across groups (LDA score > 2).

The concentrations of SCFAs in colonic contents were determined by GC-MS (7890A-5975C, Agilent Technology, Inc., Santa Clara, CA, USA). For sample preparation, colonic content samples were homogenized in a mixture of 50 μL of 15% phosphoric acid, 100 μL of an internal standard solution (125 μg/mL isocaproic acid in ether), and 400 μL of diethyl ether, followed by centrifugation at 12,000 rpm for 10 min at 4 °C. The supernatant was collected for analysis. The injection volume was 1 μL in split mode (split ratio 10:1) at an inlet temperature of 250 °C. Helium was used as the carrier gas at a flow rate of 1.0 mL/min. The oven temperature program was as follows: initial temperature 90 °C, increased to 120 °C at 10 °C/min, then to 150 °C at 5 °C/min, and finally to 250 °C at 25 °C/min with a 2 min hold. The ion source and transfer line temperatures were set at 300 °C and 250 °C, respectively. A calibration curve was constructed using a series of standard solutions containing acetic, propionic, butyric, isobutyric, valeric, isovaleric, and hexanoic acids, ranging from 0.02 to 500 μg/mL. Isocaproic acid was used as the internal standard at a fixed concentration of 25 μg/mL in all standards and samples. Quantification was based on the peak area ratio of each SCFA to the internal standard.

### 2.4. Statistical Analysis

Statistical analyses were performed using SPSS 27.0 and Origin 2025. Data are presented as the mean ± standard deviation (SD) for normally distributed variables or as the median (interquartile range) for non-normally distributed variables. Between-group comparisons were assessed by one-way analysis of variance (ANOVA) for normally distributed data with homogeneous variance, followed by the LSD post hoc test for multiple comparisons. In cases of heterogeneous variance, Tamhane’s T2 test was applied. For repeated-measures data, repeated-measures ANOVA was used. Non-normally distributed data were analyzed using the Kruskal–Wallis test. Correlation analyses were conducted using Pearson’s correlation analysis for normally distributed data and Spearman’s rank correlation analysis for non-normally distributed data. *p* < 0.05 was considered statistically significant.

## 3. Results

### 3.1. Body Weight, Body Length, and Food Utilization Rate

During the feeding period, body weight and length of animals in all groups increased significantly with time. From week 7 onward, body weight of rats in all intervention groups was significantly higher than that in the Control group. Overall, rats in the High group exhibited higher overall food utilization efficiency during the feeding period (details are shown in Figures S1–S3).

### 3.2. Effects of Milk Mineral on the Femur

**Figure 1:**
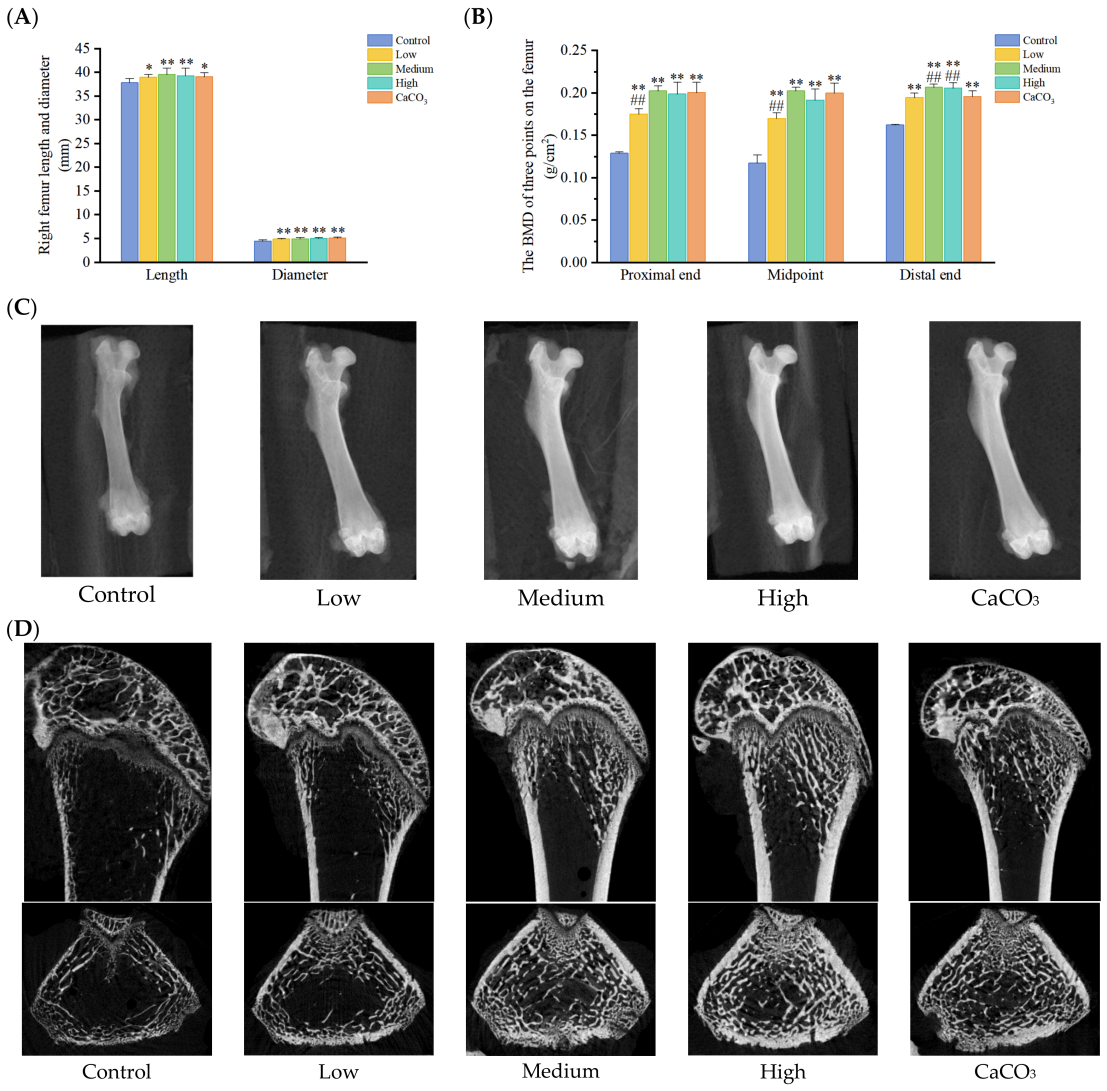

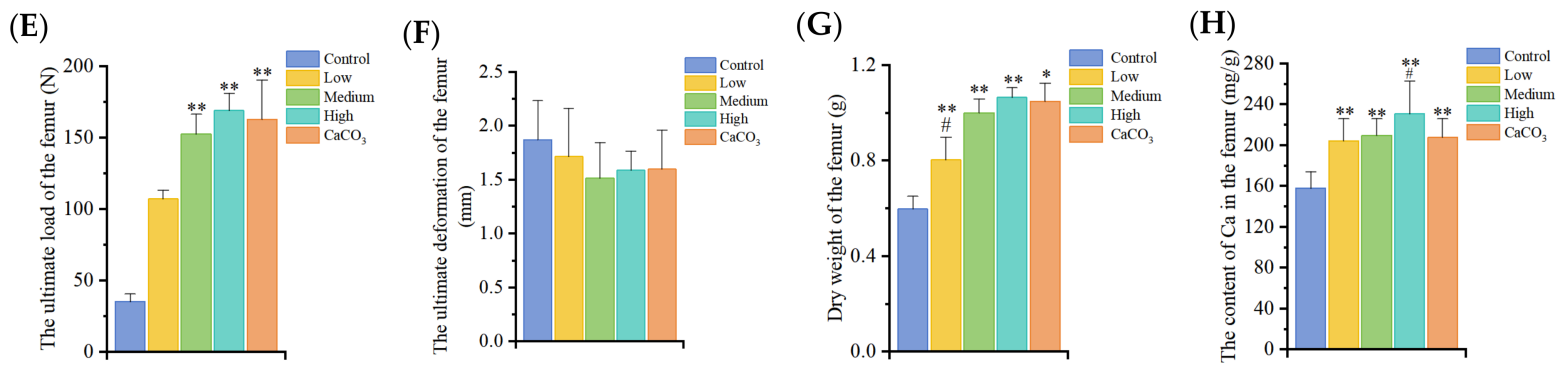
Size (**A**), BMD (**B**), representative BMD scan images (**C**), representative bone microstructure scan images (**D**), ultimate load (**E**), ultimate displacement (**F**), dry weight (**G**) and calcium content (**H**) of the right femur from each group at the end of the feeding period. Values are significantly different from the Control group at * *p* < 0.05 and ** *p* < 0.01. Values are significantly different from the CaCO_3_ group at # *p* < 0.05 and ## *p* < 0.01.

The size, BMD, and representative DXA scan results of the right femur in rats from each group at the end of the feeding period are shown in Figure 1A,C. Compared with the Control group, both the length and diameter of the right femur were significantly increased in all intervention groups (*p* < 0.05, *p* < 0.01). Compared with the Control group, all intervention groups exhibited a significant increase in BMD at the proximal, mid-point, and distal sites of the right femur (*p* < 0.01). Furthermore, the Medium and High groups demonstrated a significantly higher BMD at the distal femur compared with the CaCO_3_ group (*p* < 0.01). The microstructural parameters of the right femur for each group at the end of the feeding period are summarized in Table 2, with representative scan images presented in Figure 1D. The Medium group, despite its lower calcium content, achieved bone microstructural outcomes comparable to those of the High group and the CaCO_3_ group. Representative scan images revealed that the trabecular bone structure in the Control group was relatively sparse. In contrast, samples from the Medium, High groups, and CaCO_3_ group exhibited a more dense and well-connected trabecular network. The bone strength of the right femur in each group at the end of the feeding period is shown in Figure 1E,F. Compared with the Control group, the ultimate load in the three-point bending test was significantly increased in the Medium, High groups and CaCO_3_ group (*p* < 0.01). However, no significant differences in ultimate displacement were observed among all groups. The dry weight and calcium content of the right femur in rats from each group at the end of the feeding period are shown in Figure 1G,H. Compared with the Control group, the femoral dry weight was significantly increased in all intervention groups (*p* < 0.05, *p* < 0.01). A comparable tendency was observed for femoral calcium content, which was significantly higher in all intervention groups relative to the Control group (*p* < 0.01). Notably, the High group also demonstrated a significant increase in calcium content compared with the CaCO_3_ group (*p* < 0.05).

### 3.3. Effects of Milk Mineral on Calcium Absorption Rate

**Figure 2:**
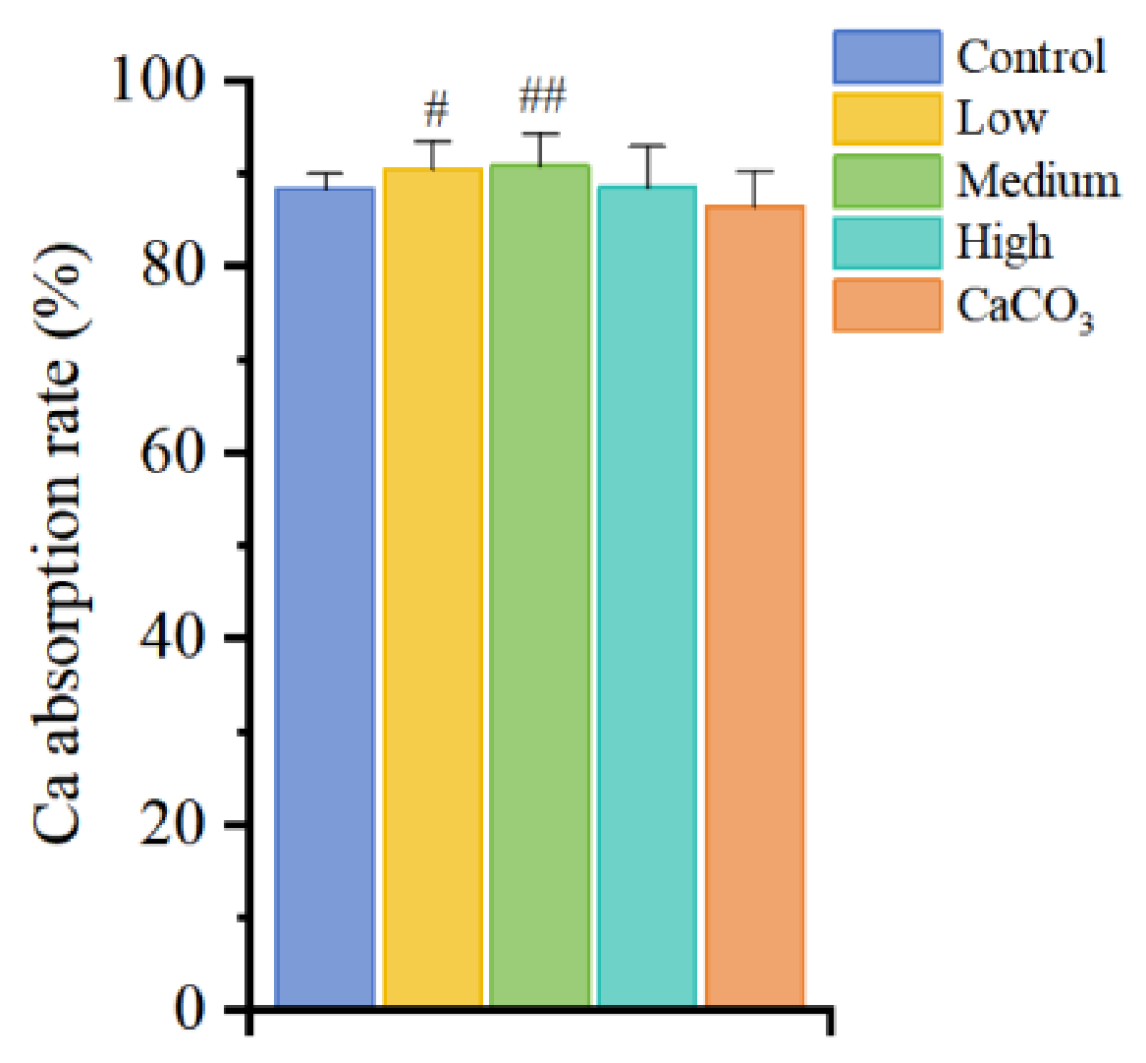
Calcium absorption rate in rats from each group. Values are significantly different from the CaCO_3_ group at # *p* < 0.05 and ## *p* < 0.01.

The calcium absorption rate in rats from each group is shown in Figure 2. Compared with the CaCO_3_ group, both the Low and Medium groups exhibited a significant increase in calcium absorption rate (*p* < 0.05, *p* < 0.01).

### 3.4. Effects of Milk Mineral on Serum Biochemical Indicators

**Figure 3:**
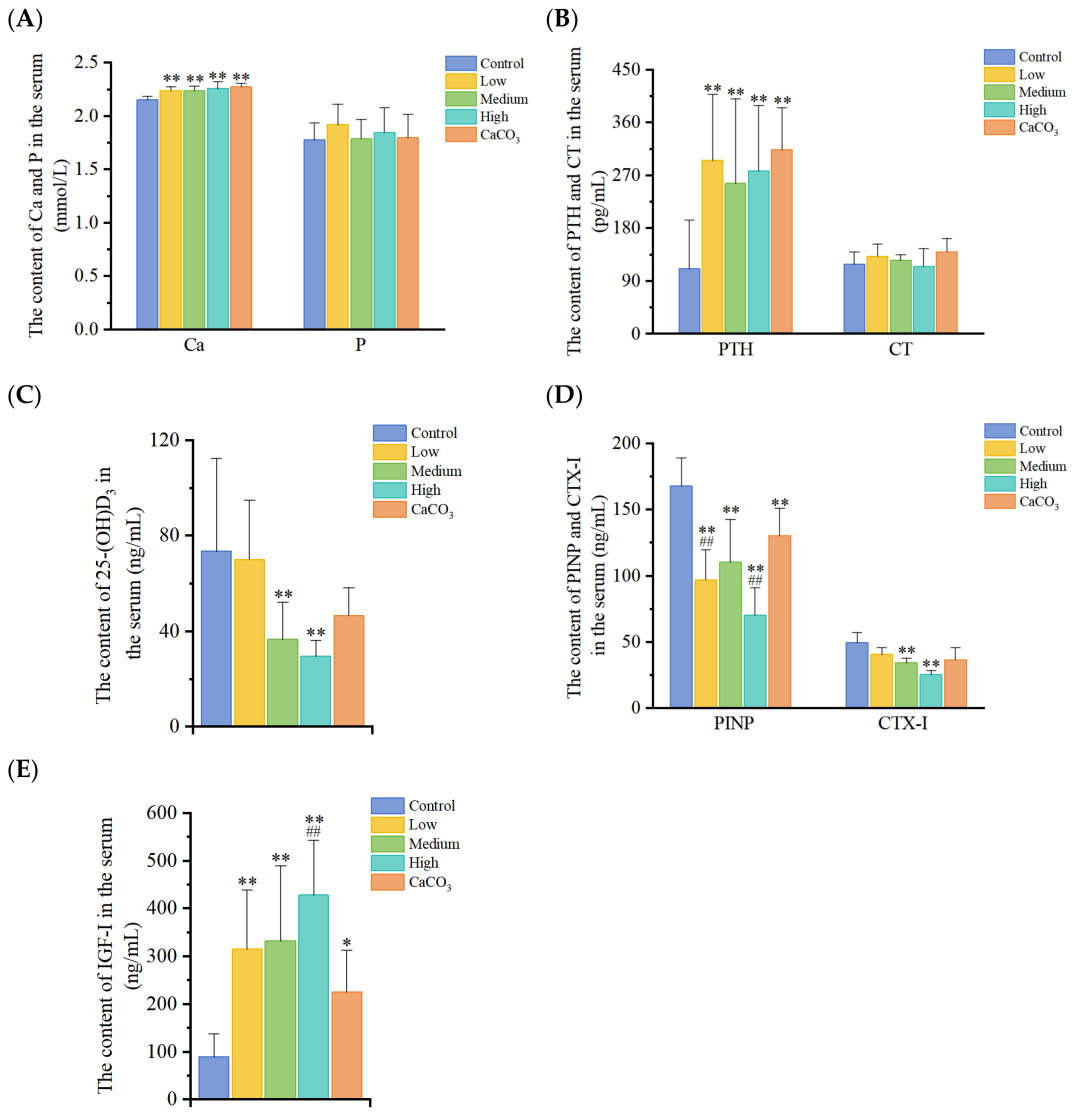
Serum biochemical indicators of bone metabolism in rats from each group at the end of the feeding period: Ca and P (**A**), PTH and CT (**B**), 25-(OH)D_3_ (**C**), PINP and CTX-I (**D**), and IGF-I (**E**). Values are significantly different from the Control group at * *p* < 0.05 and ** *p* < 0.01. Values are significantly different from the CaCO_3_ group at ## *p* < 0.01.

Serum calcium, phosphorus, PTH and CT levels (Figure 3A,B): Compared with the Control group, serum calcium and PTH levels were significantly elevated in all intervention groups (*p* < 0.01). In contrast, no significant differences in serum phosphorus and CT levels were observed among the groups. Serum 25-(OH)D_3_ levels (Figure 3C): Compared with the Control group, serum 25-(OH)D_3_ levels were significantly reduced in the Medium and High groups (*p* < 0.01). Serum PINP and CTX-I levels (Figure 3D): Compared with the Control group, serum levels of the bone formation marker PINP were significantly reduced in all intervention groups (*p* < 0.01). Furthermore, when compared with the CaCO_3_ group, the Low, and High groups all exhibited significant decreases in PINP levels (*p* < 0.05, *p* < 0.01). For the bone resorption marker CTX-I, levels were significantly lower in the High group than in the Control group (*p* < 0.01). Serum IGF-I levels (Figure 3E): Compared with the Control group, serum IGF-I levels were significantly elevated in all intervention groups (*p* < 0.05, *p* < 0.01). Moreover, the High group demonstrated a significantly higher serum IGF-I level compared with the CaCO_3_ group (*p* < 0.01).

### 3.5. Protein Expression Levels

**Figure 4:**
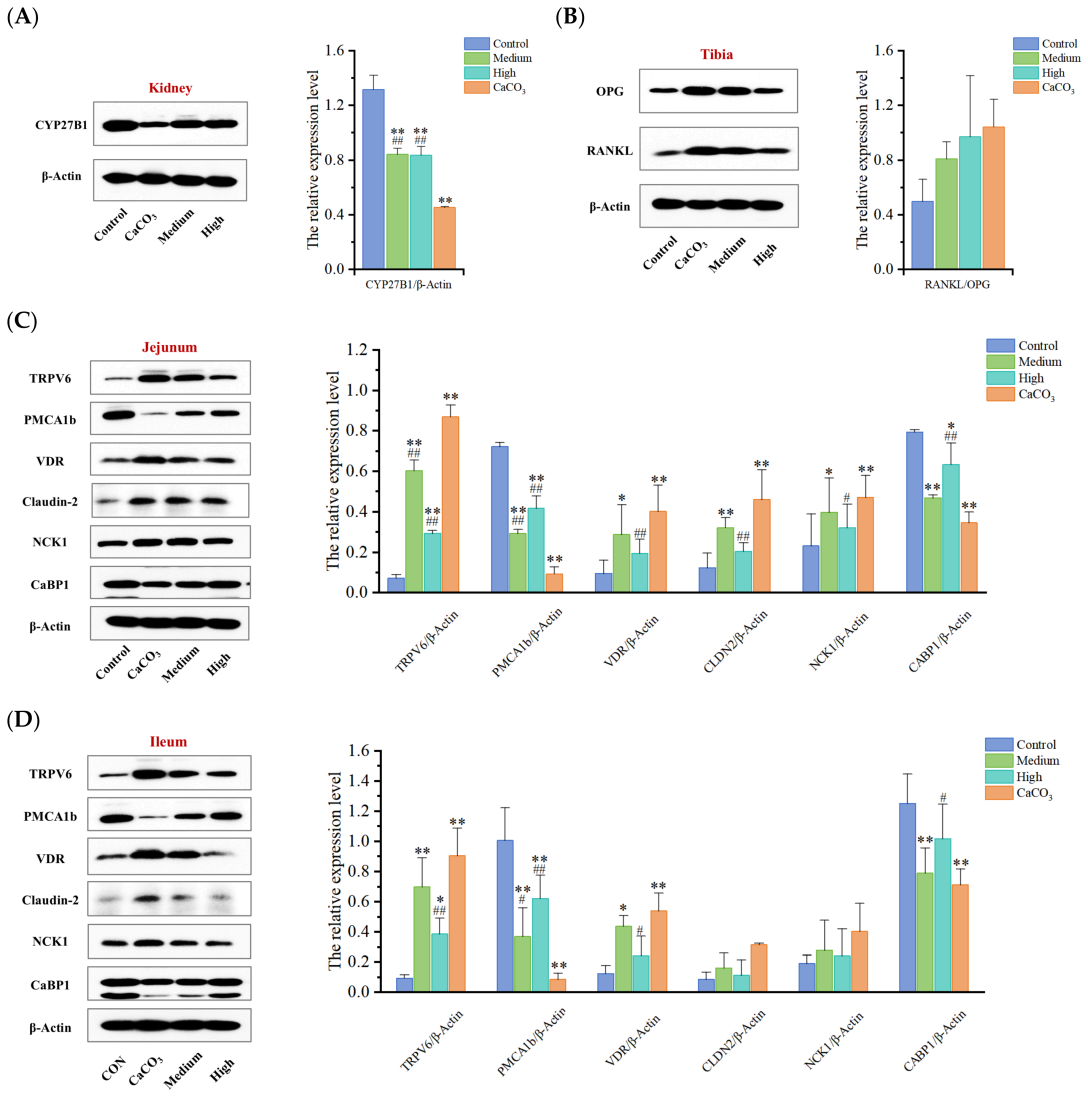

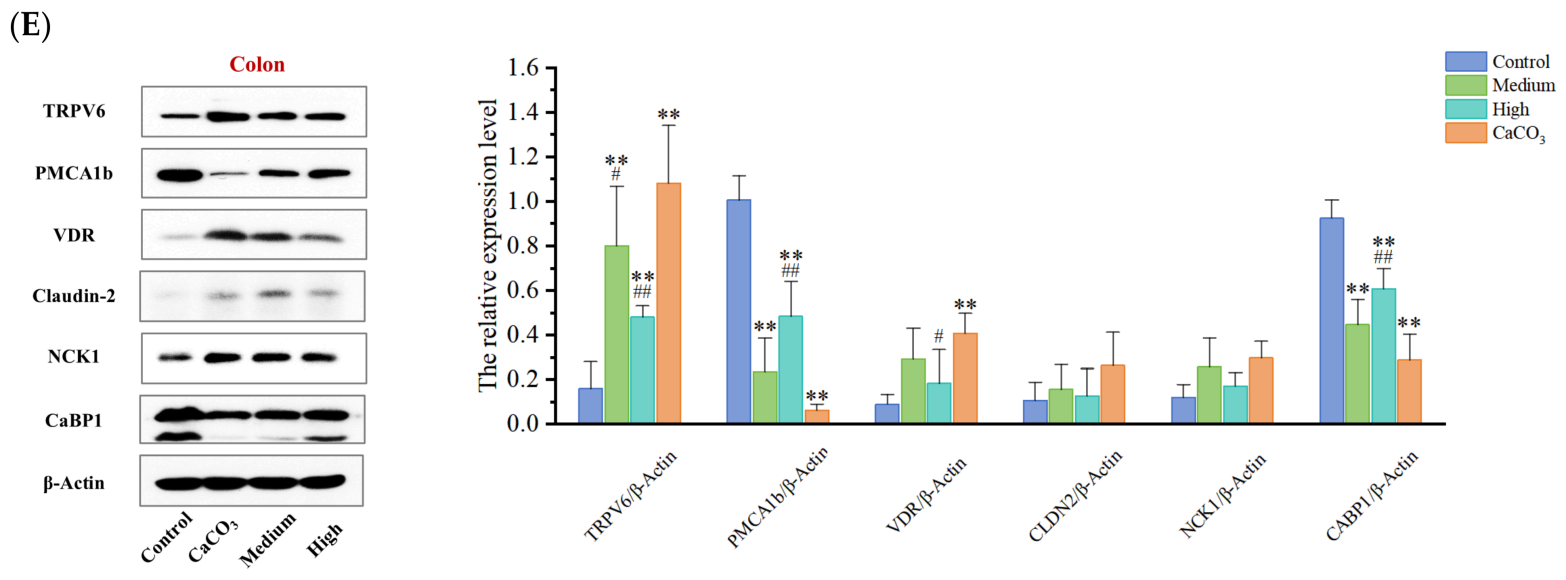
Expression level of proteins of rats from each group. Expression levels of CYP27B1 in the kidney (**A**), OPG/RANKL in the tibia (**B**), calcium absorption-related proteins in the jejunum (**C**), ileum (**D**), and colon (**E**) epithelium of rats from each group. Values are significantly different from the Control group at * *p* < 0.05 and ** *p* < 0.01. Values are significantly different from the CaCO_3_ group at # *p* < 0.05 and ## *p* < 0.01.

The expression level of CYP27B1 in the kidney of rats from each group is shown in Figure 4A. Compared with the Control group, the renal expression of CYP27B1 was significantly down-regulated in the Medium, High, and CaCO_3_ groups (*p* < 0.01). However, compared with the CaCO_3_ group, the Medium and High groups exhibited a significant up-regulation of CYP27B1 expression (*p* < 0.01). No significant differences were observed in the expression levels of either RANKL or OPG among the groups (Figure 4B). In each intestinal segment Figure 4C–E, compared with the Control group, the expression level of TRPV6 in the intestinal epithelium of rats in the Medium, High and CaCO_3_ group was significantly increased (*p* < 0.05, *p* < 0.01); whereas compared with the CaCO_3_ group, the expression levels of PMCA1b and CABP1 in the Medium and High group were significantly higher (*p* < 0.05, *p* < 0.01).

### 3.6. Gut Microbiota and SCFAs Levels

**Figure 5:**
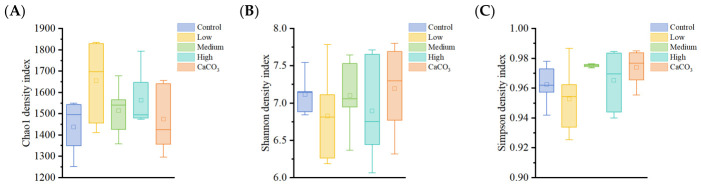

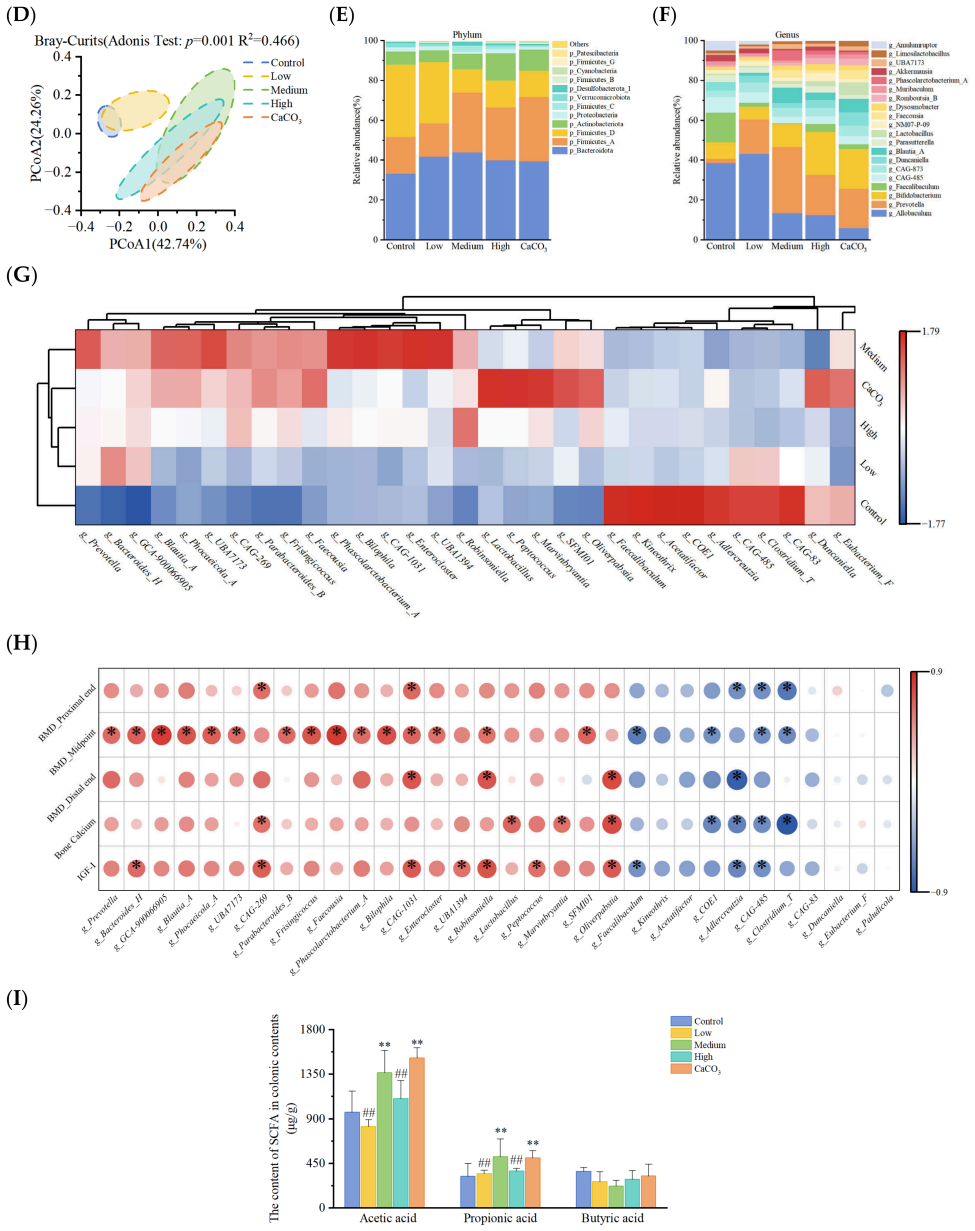
Differential analysis of gut microbiota and SCFAs in rats among all groups. Chao1 index (**A**), Shannon index (**B**), Simpson index (**C**), β diversity (**D**), relative abundance at the phylum (**E**) and genus level (**F**), grouped clustering heatmap of differential bacterial genera (**G**) and correlation heatmap between the relative abundance of differential bacterial genera and BMD, bone calcium content, and serum IGF-I levels (**H**) and concentrations of SCFAs in the colonic contents of rats from each group (**I**). In the grouped clustering heatmap, blue and red colors represent lower and higher relative abundance, respectively. In the correlation heatmap, blue and red colors indicate negative and positive correlations, respectively (* *p* < 0.05). Values are significantly different from the Control group at ** *p* < 0.01. Values are significantly different from the CaCO_3_ group at ## *p* < 0.01.

No significant differences were observed in the alpha diversity of gut microbiota among the groups (Figure 5A–D). In contrast, significant differences in β diversity were identified among the groups (*p* < 0.01). Distinct intergroup differences were observed in the relative abundance of gut microbiota at the phylum and genus level (Figure 5E,F). As shown in Figure 5G,H, the clustering patterns of differential bacterial genera in the gut microbiota were similar between the Medium group and the CaCO_3_ group. Furthermore, the relative abundances of the differentially enriched genera in the Medium and CaCO_3_ groups were generally positively correlated with femur BMD, bone calcium content, and serum IGF-I levels. In contrast, the relative abundances of the differentially enriched genera in the Control group were predominantly negatively correlated with these bone health and metabolic parameters.

As shown in Figure 5I, compared with the Control group, the concentrations of acetic acid and propionic acid were significantly increased in the Medium group and the CaCO_3_ group (*p* < 0.01).

## 4. Discussion

This study shows that the natural milk mineral exerted comparable efficacy to CaCO_3_ in enhancing bone calcium content, improving BMD, and optimizing bone microstructure in growing rats. Given that 4-week-old rats correspond roughly to childhood and adolescence in humans—a critical window for PBM acquisition—these findings suggest that milk mineral may serve as a promising nutritional strategy to support optimal bone development during growth. Notably, the Medium-dose milk mineral group achieved bone-improving effects essentially equivalent to those of the CaCO_3_ group, despite the latter having a higher calcium intake, which was likely attributable to the relatively high calcium absorption rate observed in the Medium-dose milk mineral group. Furthermore, unlike CaCO_3_, which is a single inorganic calcium source, milk mineral naturally contains bone-supporting substances such as phosphorus, magnesium, and bioactive peptides. These results not only corroborate the least non-inferior bioavailability of milk-derived calcium over inorganic calcium sources reported in prior literature [9,10], but also highlight a critical insight: the bone health-promoting effects of milk mineral may not depend solely on its elemental calcium content but are also mediated by the unique bioactive properties of its natural milk-derived matrix. However, this study did not include a CaCO_3_ group with the same calcium dosage as the Medium group, so further studies are warranted.

Bone turnover markers reflect dynamic bone metabolism balance [16]. In the present study, serum PINP levels in the Medium group were comparable to those in the CaCO_3_ group, whereas serum CTX-I levels were significantly lower in the Medium and High groups. This pattern—maintained bone formation coupled with suppressed bone resorption—indicates that long-term milk mineral supplementation helps shift bone metabolism toward a net anabolic state, consistent with previous studies [17]. This favorable balance may be explained by the coordinated regulation of multiple hormonal pathways observed in this study. Vitamin D is a well-established regulator of bone metabolism, and its deficiency has been implicated in pathological bone loss, including periodontal bone resorption [18]. Specifically, milk mineral supplementation modulated PTH secretion and upregulated renal CYP27B1—the key enzyme responsible for the activation of 25-(OH)D_3_ to 1,25-(OH)_2_D_3_—which in turn may have enhanced the conversion of 25-(OH)D_3_ to its active form, thereby promoting intestinal calcium absorption and providing adequate substrate for bone mineralization. The elevated serum IGF-I levels in milk mineral groups further reinforced this anabolic environment, as IGF-I is a well-established mediator of PTH’s anabolic effects on bone [19] and acts downstream to stimulate osteoblastic bone formation [20]. Consistent with these biochemical findings, bone mechanical tests demonstrated that milk mineral enhanced femoral stiffness, likely by promoting the deposition of inorganic bone components—a functional consequence of the improved calcium utilization and bone formation signaling described above. Collectively, these results suggest a plausible cascade in which milk mineral intake initiates enhanced calcium absorption via vitamin D pathway activation, supported by elevated IGF-I signaling that drives osteoblastic bone formation, ultimately leading to improved bone mineralization and mechanical strength. However, it should be noted that magnesium, a natural component of milk mineral, serves as an essential cofactor in the 25-(OH)D_3_ activation pathway and stabilizes bone hydroxyapatite crystals [21]. Unfortunately, serum magnesium levels were not measured in this study. Further investigations are warranted to validate the potential contribution of magnesium to these observed effects.

The classic RANKL/OPG axis dominates osteoclast differentiation and bone remodeling [22], while no significant difference in the RANKL/OPG ratio was found in this study. This finding suggests that milk mineral regulates bone metabolism independent of the RANKL/OPG pathway, and other potential regulatory mechanisms need to be explored.

Intestinal calcium absorption also significantly influences bone metabolism [23,24]. The significantly upregulated expression of intestinal CaBP1 and PMCA1b in Medium and High groups suggests enhanced capacity of TRPV6-CaBP1-PMCA1b active calcium transport system. Meanwhile, increased colonic SCFAs concentrations likely reduced intestinal pH, improved calcium solubility, and further facilitated calcium absorption, consistent with a previous observation [9]. The higher calcium absorption rate in the Medium group provided indirect support for this interpretation; however, direct confirmation via calcium flux assays or intestinal uptake experiments was not performed in this study.

The gut microbiota is another critical factor influencing bone metabolism [25,26]. In this experiment, genera such as *Lactobacillus* and *Bilophila* were enriched in the gut microbiota of rats in the Medium group and the CaCO_3_ group. These genera showed significant positive correlations with femoral BMD, bone calcium content and serum IGF-I levels. Meanwhile, previous literature has reported that microbiota-derived SCFAs may improve intestinal calcium uptake and modulate hepatic IGF-I release, which collectively could contribute to enhanced bone anabolism and mineralization [25,27]. Integrating the findings of this study, we propose a hypothetical mechanistic cascade: milk mineral may promote the enrichment of specific gut microbiota, which in turn produce SCFAs. These SCFAs are proposed to exert bone-protective effects: modulating intestinal pH to facilitate calcium absorption, and stimulating systemic IGF-I production, thereby potentially promoting bone formation, mineralization, and ultimately improving BMD. However, no significant results were observed in the functional prediction of these specific enriched genera, so their specific metabolic functions could not be determined to validate this proposed pathway. Therefore, the precise causal relationships and molecular pathways involved remain to be further elucidated in future studies.

Several limitations should be acknowledged. First, a calcium carbonate control group with the same calcium dose as the Medium group was not included, which limits direct comparison of the intrinsic effects of milk mineral at equivalent calcium levels. Second, due to technical and resource constraints, several analyses were not performed, including energy-dispersive X-ray spectroscopy (EDX) for bone mineral composition, bone histological staining and histomorphometric analysis, direct intestinal calcium absorption measurements (e.g., calcium flux assays or intestinal uptake experiments), and comprehensive bone biomechanical testing beyond femoral stiffness. These assessments would have provided additional insights into bone quality, cellular activity, and calcium transport mechanisms. Notably, species-specific differences in bone mineral composition between rodents and humans have been reported, as documented for dental tissues [28], and the absence of EDX analysis limits the assessment of how closely the bone mineral profile in this rat model reflects that of human bone. Third, serum magnesium levels were not measured, precluding evaluation of the potential contribution of magnesium to the observed bone-protective effects. Fourth, the causal relationship between gut microbiota, SCFAs, and IGF-I-mediated bone anabolism remains to be verified by targeted microbiota intervention experiments. In summary, this study provides a preliminary exploration of the potential mechanisms underlying the bone health effects of milk mineral; however, further in-depth and long-term investigations are warranted to elucidate the specific molecular mechanisms involved.

## 5. Conclusions

In growing rats, milk mineral effectively enhanced BMD, bone calcium content, and bone microarchitecture, with bone-protective effects comparable to those of calcium carbonate. Notably, the medium-dose group achieved comparable efficacy at a lower calcium intake, which may be associated with improved calcium absorption and the intrinsic bioactivity of the natural milk matrix. These bone-protective effects were accompanied by favorable changes in bone turnover markers, activation of vitamin D-related endocrine signaling, enhanced intestinal calcium transport, and enrichment of specific gut microbiota with elevated SCFA levels. Collectively, these parallel changes in bone and gut parameters suggest a possible involvement of the “gut–bone axis”, although the causal relationship remains to be established. Milk mineral may therefore represent a promising nutritional strategy to optimize PBM during growth.

## Figures and Tables

**Table 1:** Basal low-Ca diet formulation (adjusted to a Ca^2+^ content of 150 mg/100 g feed).

Ingredient	%
Casein	10.0
Soybean Meal ^1^	15.0
Wheat Flour	54.0
Corn Oil/Peanut Oil	4.0
Cellulose	2.0
Mineral Mix ^2^	2.6
Vitamin Mix ^3^	1.0
Choline Chloride	0.2
d1-Methionine	0.2
Starch ^4^	11.0

^1^ Subject to autoclaving prior to use. ^2^ Mineral mix (per kg): KH_2_PO_4_, 501.4 g; NaCl, 74.0 g; MgCO_3_, 50.2 g; ferrous lactate, 5.4 g; zinc lactate, 4.16 g; MnCO_3_, 3.5 g; CuSO_4_·5H_2_O, 0.605 g; Na_2_SeO_3_, 6.6 mg; KI, 7.76 mg; CrCl_3_·6H_2_O, 0.292 g; sucrose to 1 kg. ^3^ Vitamin mix (per kg): Vitamin A, 400,000 IU; Vitamin D_3_, 100,000 IU; Vitamin E, 500 IU; Vitamin K, 5 mg; Vitamin B_1_, 600 mg; Vitamin B_2_, 600 mg; Vitamin B_6_, 700 mg; Vitamin B_12_, 1 mg; niacin, 3 g; folic acid, 200 mg; calcium pantothenate, 1.6 g; biotin, 20 mg; sucrose to 1 kg. ^4^ The amount of starch was adjusted according to the required quantity of milk mineral (or calcium carbonate) for each experimental group.

**Table 2:** The microstructural parameters of the right femur for each group at the end of the feeding period.

Group	BV/TV (%)	SMI	Tb.Th (mm)	Tb.N (1/mm)	Tb.Sp (mm)	Conn.D (1/mm^3^)	Ct.TMD (g/cm^3^)	Ct.Th (mm)
Control	10.48 ± 2.69	1.60 ± 0.32	0.08 ± 0.01	1.30 ± 0.31	0.77 ± 0.17	64.26 ± 2.41	0.26 ± 0.02	0.22 ± 0.01
Low	14.91 ± 4.09	1.21 ± 0.14	0.13 ± 0.01 ** ##	1.46 ± 0.34 #	0.79 ± 0.07 ##	64.87 ± 15.15	0.33 ± 0.01 #	0.41 ± 0.03 #
Medium	33.72 ± 6.53 **	0.81 ± 0.39 **	0.13 ± 0.01 **	2.61 ± 0.47 *	0.41 ± 0.14	104.01 ± 18.75 **	0.36 ± 0.02 **	0.52 ± 0.01 *
High	37.93 ± 8.15 **	0.93 ± 0.49 **	0.14 ± 0.00 ** #	2.58 ± 0.67 *	0.40 ± 0.19 *	112.19 ± 13.49 **	0.36 ± 0.01 **	0.55 ± 0.02 **
CaCO_3_	34.70 ± 3.78 **	1.01 ± 0.42 **	0.13 ± 0.01 **	2.87 ± 0.31 **	0.29 ± 0.05 **	139.50 ± 27.15 **	0.36 ± 0.01 **	0.53 ± 0.04 **

BV/TV: bone volume/tissue volume; SMI: structure model index (indicating the ratio of plate-like to rod-like structures in trabecular bone); Tb.Th: trabecular thickness; Tb.N: trabecular number; Tb.Sp: trabecular separation; Conn.D: connectivity density, representing the number of connections per cubic millimeter within the trabecular network; Ct.TMD: cortical bone tissue mineral density; Ct.Th: cortical thickness. Significantly different from the Control group at * *p* < 0.05 and ** *p* < 0.01. Significantly different from the CaCO_3_ group at # *p* < 0.05 and ## *p* < 0.01.

## Data Availability

The datasets used during the current study are available from the corresponding author upon reasonable request.

## Supplementary Materials

The following supporting information can be downloaded at: https://www.mdpi.com/article/10.3390/nu18152569/s1, Figure S1: Body weight of rats in each group during the feeding period; Figure S2: Body length of rats in each group during the feeding period; Figure S3: Food utilization rate of rats in each group during the feeding period.

## Abbreviations

Full Term	Abbreviation
Bone mineral density	BMD
bone volume fraction	BV/TV
Calcium	Ca
Calbindin-D9k	CaBP1
Calcium carbonate	CaCO_3_
Claudin-2	CLDN2
connectivity density	Conn.D
Calcitonin	CT
cortical thickness	Ct.Th
cortical tissue mineral density	Ct.TMD
C-terminal telopeptide of type I collagen	CTX-I
Cytochrome P450 Family 27 Subfamily B Member 1	CYP27B1
Dual-energy X-ray Absorptiometry	DXA
Insulin-like Growth Factor-1	IGF-I
milk mineral concentrate	MMC
NCK Adaptor Protein 1	NCK1
Osteoprotegerin	OPG
Phosphorus	P
Peak bone mass	PBM
Procollagen Type I N-Terminal Propeptide	PINP
Plasma Membrane Calcium ATPase 1b	PMCA1b
Parathyroid hormone	PTH
Receptor Activator of Nuclear Factor Kappa-B Ligand	RANKL
short-chain fatty acid	SCFA
structure model index	SMI
specific pathogen-free	SPF
trabecular number	Tb.N
trabecular separation	Tb.Sp
trabecular thickness	Tb.Th
Transient Receptor Potential Cation Channel Subfamily V Member 6	TRPV6
vitamin D receptor	VDR
