# Supplementary material for: Mechanistic Insights into Milk Minerals Driving Bone Development and Mineralization in Growing Rats
Source: Nutrients. 2026 Aug 6;18(15):2569. doi: 10.3390/nu18152569 (PMC13468487; doi:10.3390/nu18152569)
Supplement: Supplementary file 1 [file nutrients-18-02569-s001.zip › nutrients-4402196-supplementary.pdf]

Supplementary Materials

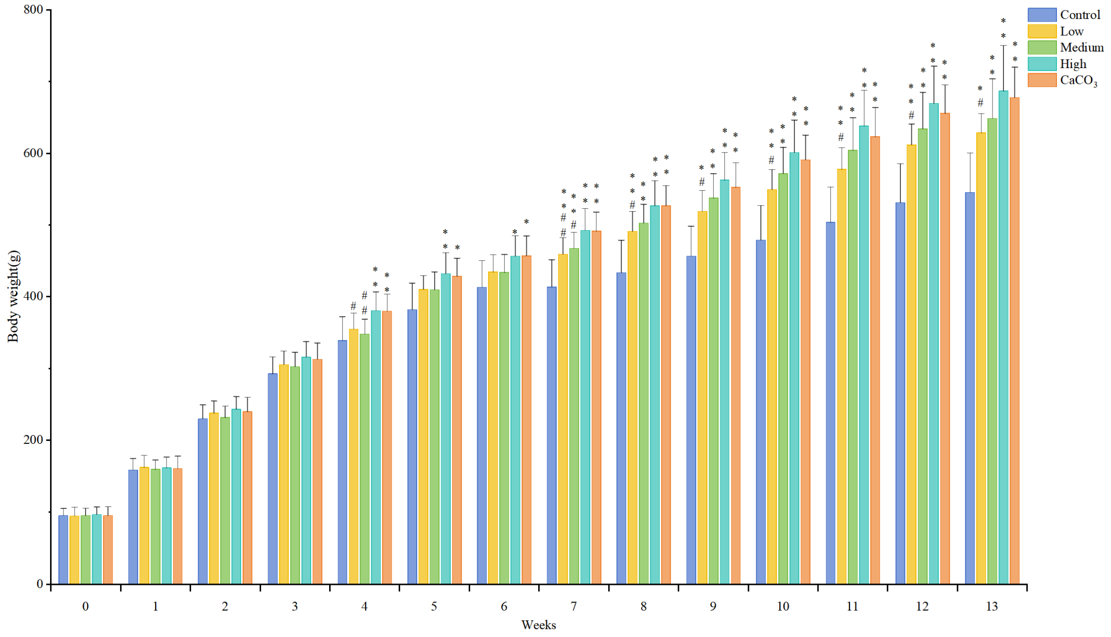

Figure S1. Body weight of rats in each group during the feeding period. Values are significantly different from the Control group at *p < 0.05 and **p < 0.01. Values are significantly different from the $CaCO_3$ group at #p < 0.05 and ##p < 0.01.

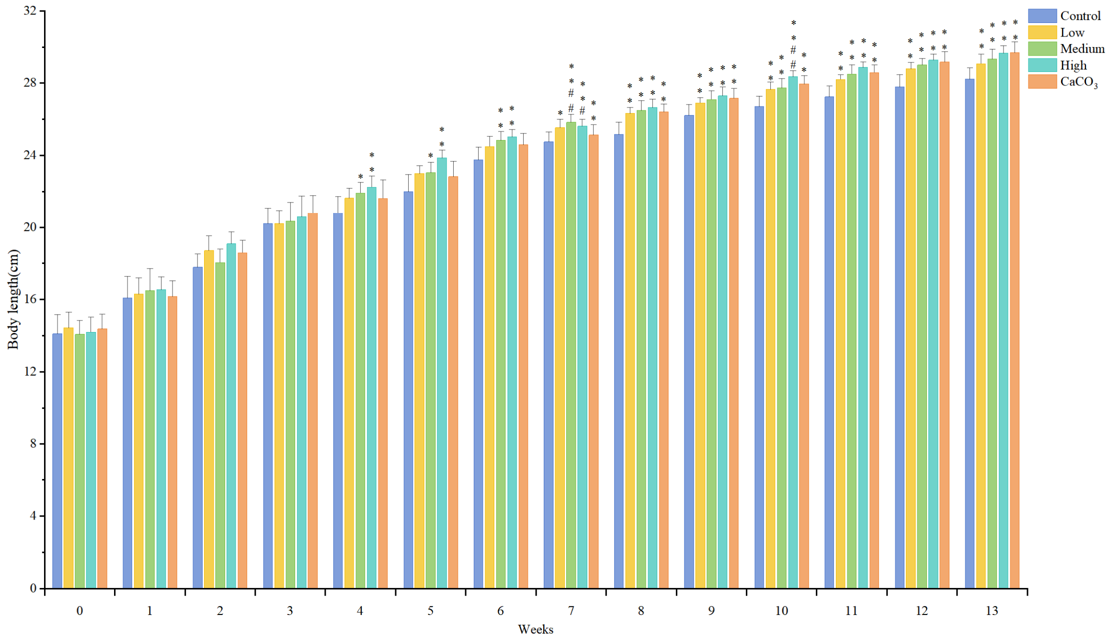

Figure S2. Body length of rats in each group during the feeding period. Values are significantly different from the Control group at *p < 0.05 and **p < 0.01. Values are significantly different from the $CaCO_3$ group at #p < 0.05 and ##p < 0.01.

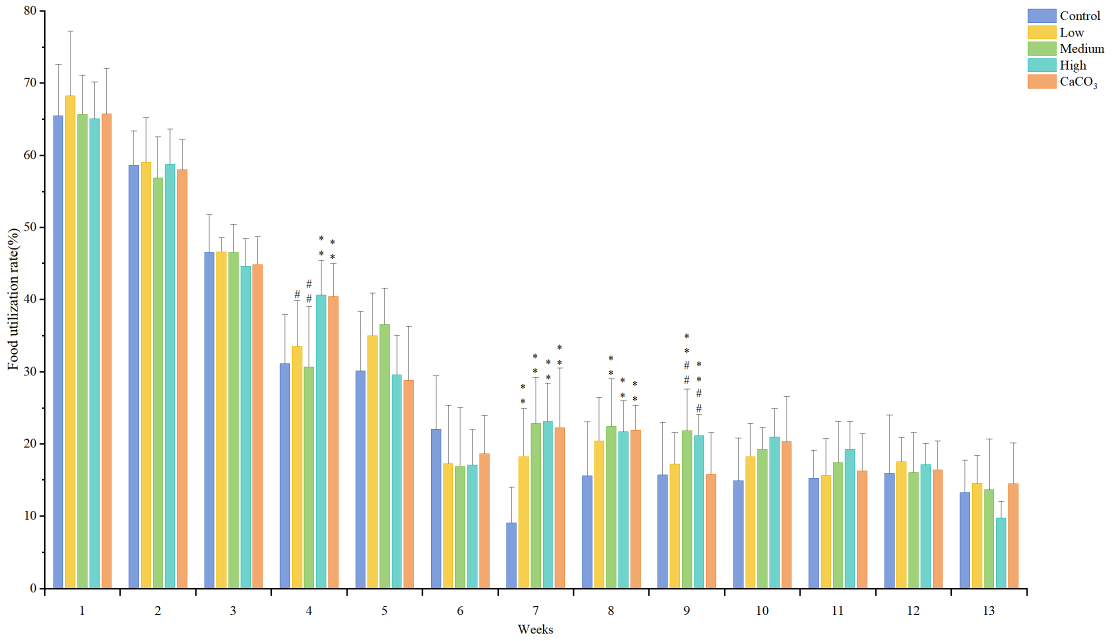

Figure S3. Food utilization rate of rats in each group during the feeding period. Values are significantly different from the Control group at $^{**}p < 0.01$. Values are significantly different from the $CaCO_3$ group at $\#p < 0.05$ and $\#\#p < 0.01$.
